# Long-Term Outcomes After Chemoradiotherapy and Surgery for Superior Sulcus Tumors

**DOI:** 10.1016/j.jtocrr.2023.100475

**Published:** 2023-02-24

**Authors:** S. Ünal, J.A. Winkelman, D.J. Heineman, I. Bahce, M. van Dorp, J.A. Braun, S. Hashemi, S. Senan, M.A. Paul, M. Dahele, C. Dickhoff

**Affiliations:** a Department of Cardiothoracic Surgery, Amsterdam UMC location Vrije Universiteit Amsterdam, Cancer Center Amsterdam, Amsterdam, The Netherlands; b Department of Pulmonary Medicine, Amsterdam UMC location Vrije Universiteit Amsterdam, Cancer Center Amsterdam, Amsterdam, The Netherlands; c Department of Cardiothoracic Surgery, Leiden University Medical Center, Leiden, The Netherlands; d Department of Radiation Oncology, Amsterdam UMC location Vrije Universiteit Amsterdam, Cancer Center Amsterdam, Amsterdam, The Netherlands

**Keywords:** Superior sulcus, Pancoast tumor, Non–small cell lung cancer, Clinical outcomes, Pathologic response, Trimodality therapy

## Abstract

**Introduction:**

Superior sulcus tumors (SSTs) are uncommon, and their anatomical location can make treatment challenging. We analyzed late outcomes of patients with SST treated with concurrent chemoradiotherapy followed by surgical resection (trimodality) in a single tertiary institution.

**Methods:**

Patients with non–small cell SSTs, who underwent trimodality therapy between 2002 and 2017, were selected from a prospective institutional surgical database. Patients were uniformly staged with 18F-fluorodeoxyglucose–positron emission tomography, computed tomography scan of the chest and upper abdomen, and brain imaging. Patients undergoing resection of the lung plus chest wall were grouped as limited SST and those needing extensive resections (e.g., including the vertebral body) as extended SST. Kaplan-Meier survival analysis was performed to determine difference in survival. Multivariate Cox regression was used to identify prognostic factors.

**Results:**

A total of 123 patients were identified with a median follow-up of 4.9 years (interquartile range: 1.6–8.9 y). The 90-day postoperative mortality and morbidity (Clavien-Dindo grades III–V) were 6.5% and 21.1%, respectively. Patients with a radical resection (R0: 92.7%) had better survival (*p* = 0.002), as did those who had major pathologic response (73%) (*p* = 0.001). Ten-year overall survival (OS) and disease-free survival were 48.1% and 42.6%, respectively. There were no differences in 90-day mortality (*p* = 0.31) and OS (*p* = 0.79) between extended SST and limited SST patients.

**Conclusions:**

In patients with SST, trimodality resulted in a 10-year estimated OS and disease-free survival of 48.1% and 42.6%, respectively, which were improved after radical resection (R0) and major pathologic response. Survival for limited and extended resections was comparable, and distant relapse was the main pattern of failure. Better systemic treatments are therefore needed.

## Introduction

For medically and technically operable patients with NSCLC tumor located in the superior sulcus, induction chemoradiotherapy (CRT) followed by surgery is the recommended treatment.^1^ The recommendation for the so-called trimodality therapy is largely based on the results of the prospective multicenter Southwest Oncology Group Trial 9416 and Japanese JCOG 9806 trials, in which complete removal of the tumor resulted in a 5-year overall survival (OS) of 54% and 70%, respectively.^2^^,^^3^ Nevertheless, surgery for superior sulcus tumors (SSTs) is generally considered challenging because of their location in the apex of the lung with involvement of the chest wall and to dense fibrosis and adhesions resulting from induction CRT. Once additional surrounding structures are involved, such as the vertebrae or large vessels (superior caval vein or subclavian artery), complete and safe resections may be even more difficult and may be a reason to proceed with definitive CRT.

Data on clinical outcomes are important to justify the use of complex pulmonary resections for SST. In addition, real-world data from large contemporary series can be used to benchmark results of ongoing studies, both with or without use of surgery. In this article, we explored perioperative mortality and long-term survival in a large cohort of patients, uniformly staged (including fluorodeoxyglucose–positron emission tomography [FDG-PET] scan and imaging of the brain) and treated (with guideline-recommended trimodality therapy), with all operations performed by surgeons from a single tertiary referral center. Second, we focus on the outcomes of patients who needed an extended resection.

## Material and Methods

### Study Design and Patient Selection

After approval of the Institutional Medical Ethics Committee (approval number 2021.0635), patients with pathology-proven, nonmetastasized non–small cell lung tumors with radiological involvement of the thoracic wall above the second rib, and treated with trimodality therapy between 2002 and 2017, were selected from a prospective surgical database. From 2003, the standard institutional approach for SST was based on the recommendations of the International Association for the Study of Lung Cancer.^4^ Patients were operated on at a single tertiary referral center that treats approximately nine patients with SST/y and is the highest volume center for SST surgery in the Netherlands.^5^ For logistic reasons, a few patients received surgery in the referring hospital, but the surgery was performed by the tertiary surgical team. After uniform staging, using contrast-enhanced thoracic/upper abdominal computed tomography (CT) scan, whole-body 18F-FDG-PET(/CT) scan, and imaging of the brain, resectability was discussed in a multidisciplinary tumor board including thoracic surgeons experienced in complex thoracic surgery. In patients with suspected nodal involvement (cN2/3) on CT or PET(/CT) scan, staging included endoscopic ultrasound, endobronchial ultrasound, or (video)mediastinoscopy. Imaging of the brain was standard from 2007, initially with CT and then subsequently with magnetic resonance imaging. For the purpose of the current study, patients initially staged with the sixth edition of the TNM were restaged according to the seventh edition. To analyze the impact of extended surgery on outcome, we defined two groups. The first group consisted of patients with SST treated with standard pulmonary resection and en-bloc removal of a part of the chest wall (limited SST [L-SST]). The second group consisted of those patients in whom an extended resection was performed, that is, additional bronchial or arterial sleeve resection and resection of the subclavian artery, pedicle, or body of the vertebra, or the sternum (extended STT [E-SST]).

### Trimodality

If patients were medically operable, and the tumor was considered technically resectable, they were scheduled for induction CRT at the tertiary institute or referring hospital. Chemotherapy typically consisted of one cycle of cisplatin plus gemcitabine before the start of radiotherapy, followed by two cycles of cisplatin plus etoposide concurrent with radiotherapy for patients with squamous cell histology and NSCLC not otherwise specified (NSCLC-NOS). For those with adenocarcinoma, three cycles of cisplatin and etoposide concurrent with radiotherapy concurrent from day 1 of the first or second cycle of chemotherapy was standard until 2016, but based on the results of the PROCLAIM study, etoposide was then replaced by pemetrexed.^6^ The currently, and most often, used radiation schedule are 25 fractions of 2 Gy for tumors judged to be resectable upfront and 30 fractions of 2 Gy for tumors considered to be borderline resectable. Reassessment after CRT was performed with CT scan of the chest and upper abdomen and a whole-body FDG-PET(/CT) scan approximately 3 to 4 weeks after the last day of radiotherapy. Tumor response was measured using Response Evaluation Criteria in Solid Tumors and discussed in the multidisciplinary tumor board.^7^ Patients proceeded to surgery in the absence of disease progression, and resection was performed 5 to 6 weeks after completion of CRT.^8^ If there was clinically suspicious, or pathological proven mediastinal nodal involvement before the start of induction treatment, and no radiological progression postinduction, the patient proceeded to surgery without invasive restaging, because limited persistent mediastinal nodal involvement was not a contraindication for resection. Anatomical pulmonary resection was performed as standard, except for patients with limited pulmonary reserve, in whom a wedge resection could be considered. The Clavien-Dindo classification for grading complications after surgery was used to evaluate postoperative morbidity.^9^ Pathologic complete response (pCR) was defined as no viable tumor and major pathologic response (MPR) as less than or equal to 10% residual viable tumor present within the primary tumor bed. A follow-up chest/upper abdominal CT scan was planned every 3 months for the first 2 years after surgery, every 6 months in years 2 to 5, and yearly thereafter, according to national guidelines.^10^ In the event of missing data, the referring physician or general practitioner was contacted.

### Outcome and Statistical Analysis

OS was defined as the time between surgery and date of death or last date of follow-up (October 1, 2020). The disease-free survival (DFS) was defined as the time between surgery and date of (locoregional or distant) recurrence, secondary primary lung cancer (SPLC), or death in the absence of disease progression. Locoregional recurrence was defined as objective tumor progression or relapse in the area of previous surgery or locoregional lymph nodes (N1–2). Normally distributed continuous variables were presented as means and SD, non-normally distributed data by their median and interquartile range (IQR). Categorical variables were presented as frequencies with percentages. Normally distributed continuous data were tested with the independent-sample Student’s *t* test. Non-normally distributed data were tested with the Mann-Whitney *U* test. Categorical variables were tested using the Pearson’s chi-square test or Fisher’s exact test as appropriate. The Kaplan-Meier method was used for 2-, 5-, and 10-year survival rates. Potential prognostic factors for OS and DFS were assessed using Cox regression analysis in which a *p* value of less than 0.10 was considered significant for factors in univariate analysis, and statistical significant factors in univariate analysis were tested in multivariate analysis using backward selection in which a *p* value of less than 0.05 was considered significant. Categorical characteristics were assessed by chi-square or Fisher’s exact test. All analyses were conducted using SPSS statistics (version 26.0).

## Results

### Patients and Treatment

**Figure 1:**
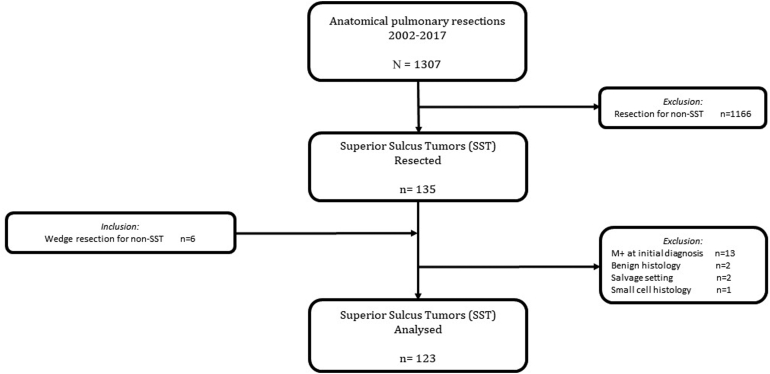
Cohort diagram of the study population.

From our institutional surgical database, we identified 141 patients with SST, of whom 13 patients were diagnosed with having metastasis before start of therapy, two patients with an inflammatory pseudotumor, two patients were operated on in a salvage setting, and one patient had small cell histology, resulting in 123 patients eligible for analysis (77 men, 46 women), with a mean age of 56.6 years (SD 9.4 y) (Fig. 1). Clinical stages were IIB (n = 46), IIIA (n = 70), and IIIB (n = 7). Histologic subtypes were NSCLC-NOS (n = 51), adenocarcinoma (n = 42), and squamous cell carcinoma (n = 30). Planned radiotherapy was completed in 118 (95.9%) of patients, and chemotherapy dose was reduced by 25% in four patients and 50% in one patient. Concurrent CRT consisted of a total radiotherapy dose of 60 to 66 Gy in 30 patients (24%), 50 to 59 Gy in 66 patients (54%), and 39 to 49 Gy in 27 patients (22%). All patients had a restaging PET(/CT) scan, planned approximately 3 weeks after the last day of radiotherapy. Demographics and clinical characteristics are summarized in Table 1.

**Table 1:** Demographic and Clinical Characteristics of Patients (n = 123) With Superior Sulcus Tumors, Treated With Chemoradiotherapy and Surgery Between 2002 and 2017

Demographic and Clinical Characteristics	n	%
Number of patients	123	
Sex (male:female)	77 vs. 46	
Age (mean, SD)	56.6 (9.4)	
Mediastinal nodal evaluation		
EUS	16	13.0
EBUS	24	19.5
Mediastinal	15	12.2
Radiotherapy		
39–49 Gy	27	22.0
50–59 Gy	66	53.7
60–66 Gy	30	24.4
Reduction in dose radiotherapy		
0%	118	95.9
25%	4	3.3
50%	1	0.8
Tumor characteristics		
Histopathology		
NSCLC-NOS	51	41.5
Adenocarcinoma	42	34.1
Squamous cell carcinoma	30	24.4
Laterality		
Right	92	74.8
Left	31	25.2
Clinical T status		
T3	76	61.8
T4	47	38.2
cTNM seventh edition (2010–2018)		
IIb	46	37.4
IIIa	70	56.9
IIIB	7	5.7
Clinical nodal status		
No lymph nodes	83	67.5
Hilar	19	15.4
Mediastinal	20	16.3
Supraclavicular	1	0.8
Surgical characteristics		
Surgical approach		
Anterior	12	9.8
Posterolateral	110	89.4
Combined	1	0.8
Type of pulmonary resection		
Lobectomy	108	87.8
Bilobectomy	2	1.6
Wedge resection	6	4.9
Lobectomy and wedge	3	2.4
Segmentectomy	2	1.6
Sleeve	2	1.6
Extended resection		
Subclavian artery	2	1.6
Bronchial sleeve	1	0.8
Combined bronchial/arterial sleeve	1	0.8
Complete vertebra(e)	6	4.9
Partial vertebra(e)	4	3.2
Partial sternectomy + partial vertebra(e)	1	0.8
Partial sternectomy + subclavian artery	2	1.6
Resection chest wall (number of ribs)		
None	1	0.8
One	5	4.1
Two	16	13.0
Three	42	34.1
Four	51	41.5
Five	8	6.5
Pathology characteristics		
Resection margin		
R0	114	92.7
R1	9	7.3
Pathologic response rate		
Major (complete) response	84 (52)	68.3 (42.3)
Nonmajor response	39	31.7
pTNM (seventh edition 2007–2018)		
0	52	42.3
Ia	23	18.7
IIa	2	1.6
IIb	37	30.1
IIIa	9	7.3

cTNM, clinical TNM; EBUS, endobronchial ultrasound; EUS, endoscopic ultrasound; NSCLC-NOS, NSCLC not otherwise specified; pTNM, pathologic TNM.

Patients underwent en-bloc lung and chest wall resection through an anterior (n = 12), posterolateral (n = 110), or combined (n = 1) approach. The type of surgical resection was lobectomy (n = 108), bilobectomy (n = 2), lobectomy plus wedge resection (n = 3), sleeve lobectomy (n = 2), segmentectomy (n = 2), or wedge resection (n = 6). The 90-day postoperative morbidity was 21.1% (Clavien-Dindo grade III n = 16, grade IV n = 8, and grade V n = 2). Radical resection (R0) was achieved in 114 patients (92.7%) and the remaining nine patients (7.3%) had a microscopically incomplete (R1) resection. There were 17 patients in the E-SST group based on additional resection of the vertebra (partial n = 4, complete n = 6), a bronchial sleeve (n = 1), combined bronchial/arterial sleeve (n = 1), partial sternectomy and partial vertebral resection (n = 1), partial sternectomy and subclavian artery (n = 2), or subclavian artery (n = 2). A MPR was found in 84 patients (68.3%), of which 52 had pCR (42.3%). Pathologic tumor stages were as follows: ypT0N0 (n = 52), ypT0N1 (n = 1), ypT1N0 (n = 23), ypT2N0 (n = 1), ypT2N1 (n = 1), ypT3N0 (n = 36), ypT3N1 (n = 2), ypT3N2 (n = 1), and ypT4N0 (n = 6).

### Mortality and Survival

**Figure 2:**
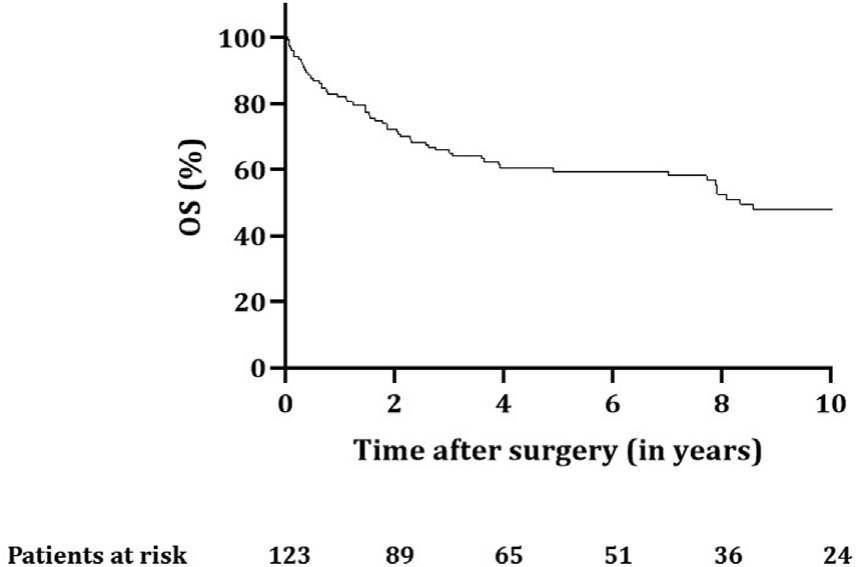
OS for all patients (n = 123) with SST treated with chemoradiotherapy and surgery. OS, overall survival; SST, superior sulcus tumor.

**Figure 3:**
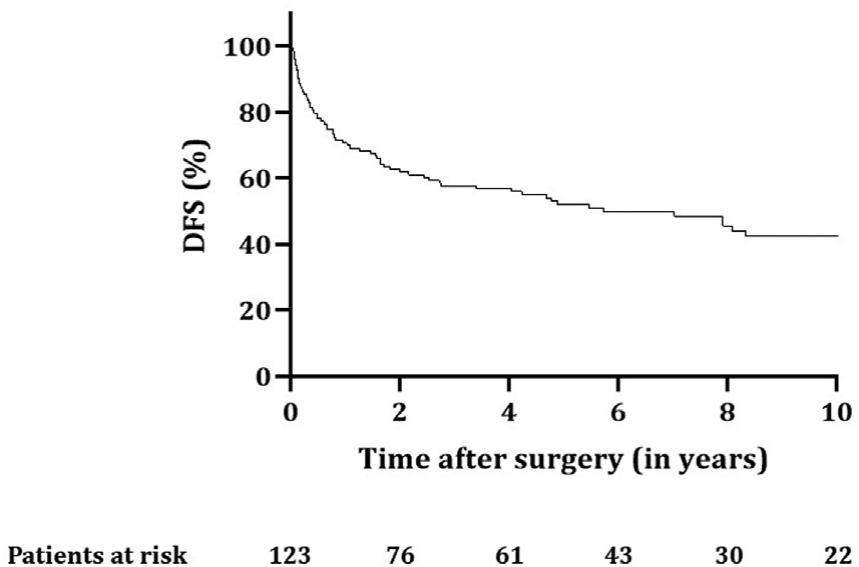
DFS for all patients (n = 123) with SST treated with chemoradiotherapy and surgery. DFS, disease-free survival; SST, superior sulcus tumor.

Follow-up was complete for all patients up to October 1, 2020. Median follow-up was 4.9 (IQR: 1.6–8.9) years. The 90-day postoperative mortality rate was 6.5% (n = 8): six patients died of disease progression, one because of massive bleeding 35 days after surgery, and one patient died from a ruptured abdominal aortic aneurysm 35 days after surgery. OS rates for all patients at 2, 5, and 10 years were 72.4%, 59.5%, and 48.1%, respectively (Fig. 2), with a median of 8.3 years (confidence interval [CI]: 5.7–10.9). The 2-, 5-, and 10-year DFS rates were 61.8%, 52.1%, and 42.6%, respectively, with a median of 5.7 years (CI: 2.6–8.8) (Fig. 3).

Of the patients who died during follow-up (n = 63), 52 deaths were disease related (82.5%). Tumor recurrence occurred in 52 of 123 patients, predominantly (83.3%) within the first 3 years after surgery. Locoregional recurrence without metastases occurred in six patients (4.9%), distant metastases in 35 patients (28.5%), and both local recurrence and distant metastases in 11 patients (8.9%). Distant metastases were found in the brain (n = 20), contralateral lung (n = 6), bone (n = 5), bone and adrenal gland (n = 5), brain and bone (n = 5), adrenal glands only (n = 3), liver (n = 2), brain and kidney (n = 1), and stomach (n = 1). An SPLC was diagnosed in four patients (3.3%), and an SPLC with distant metastases in two patients (1.6%).

**Figure 4:**
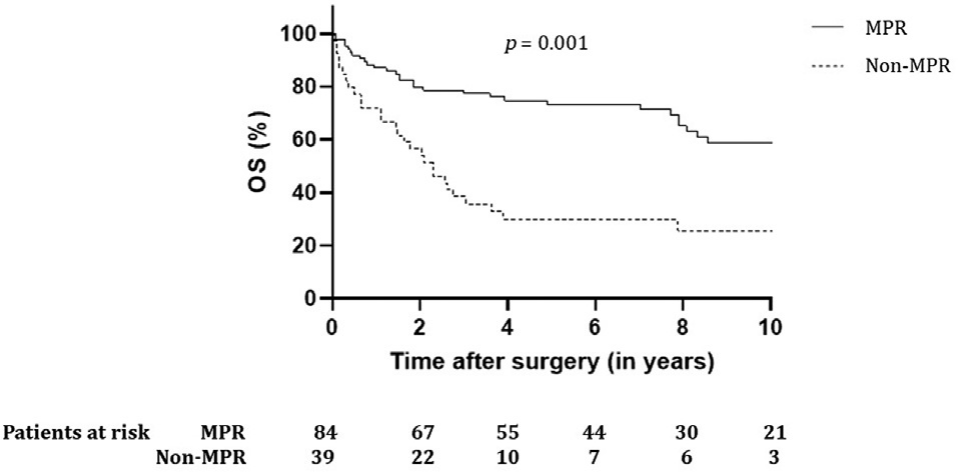
OS for patients with SST with major (including complete) versus non-MPR. MPR, major pathologic response; OS, overall survival; SST, superior sulcus tumor.

Results of univariate and multivariate Cox regression analyses of potential predictors for OS and DFS are presented in Supplementary Table 1. Univariate analysis revealed R1 resection and MPR (including pCR) as prognostic for OS. After multivariate testing, only MPR remained significant (hazard ratio [HR] = 0.37, CI 0.22–0.65, *p* < 0.001). Incomplete resections were more often found in non-MPR (n = 8) versus MPR (n = 1, *p* < 0.001). Of patients with complete resection, 62.5% were alive after 5 years compared with 14.3% of patients with R1 resection (*p* = 0.002). Patients with MPR (including pCR) had improved 5-year survival when compared with patients with no MPR: 73.3% versus 29.8% (*p* = 0.001), which is found in Figure 4 (HR = 0.60, CI: 0.34–1.05, *p* = 0.08). Univariate analysis revealed MPR and R1 resections as prognostic for survival, of which only MPR remained significant when tested in multivariate analysis (HR = 0.37, CI: 0.22–0.61, *p* < 0.001). The 5-year DFS for patients with R1 resection was 11.1% compared with 55.5% for patients with R0 (*p* = 0.002), and DFS at 5 years for patients with MPR was significantly better than that for patients with non-MPR: 67.3% versus 19.2% (*p* < 0.001). No difference in survival was found between T3 and T4 tumors.

### Limited Versus Extended SST

There were 106 patients in the L-SST group and 17 in the E-SST group. Comparison of characteristics between L-SST and E-SST can be found in Supplementary Table 2. Median follow-up time for L-SST and E-SST was 4.96 (IQR: 1.7–9.0) years and 3.64 (IQR: 1.0–9.8) years, respectively. When compared with L-SST, patients in the E-SST group were younger (mean age 51 versus 57 y) and had less clinical involvement of the lymph nodes at baseline staging (11.8% versus 35.8%, *p* = 0.049). Complete surgical resection rate was 94.1% in the E-SST group and 92.5% in the L-SST group (*p* = 1.0), and MPR (including pCR) response rates was 76.5% for E-SST and 57.7% (*p* = 0.44) for L-SST.

The 90-day mortality was 11.8% (two of 17) for E-SST and 5.7% for L-SST patients (*p* = 0.31).

**Figure 5:**
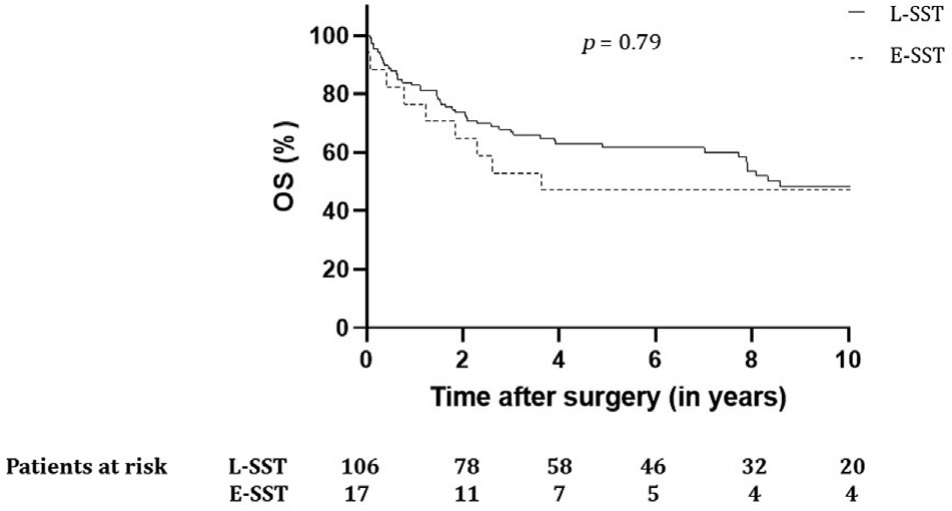
OS for patients with L-SST and E-SST treated with chemoradiotherapy and surgery. E-SST, extended superior sulcus tumor; L-SST, limited superior sulcus tumor; OS, overall survival.

Disease relapse occurred in nine patients (52.9%) in the E-SST group compared with 43 patients (40.6%) in the L-SST group. Distribution of recurrence in the E-SST group was locoregional in two, distant in six, and both locoregional and distant in one. In the L-SST group, four patients with SPLC were found, two patients with SPLC and synchronous metastases, four patients with locoregional recurrence, 29 patients with metastases, and 10 patients had both locoregional recurrence and metastases. Mean OS was 9.0 years (CI 7.5–10.5) in the L-SST group compared with 9.2 years (CI 5.3–13.1) in the E-SST group. OS rates for 2, 5, and 10 years were 73.6%, 61.6%, and 48.5% for L-SST and 64.7%, 47.1%, and 47.1% for E-SST (*p* = 0.79) (Fig. 5). Median DFS was 7.90 years (4.8–11.0) for L-SST and 3.40 years (0.0–7.7) for the E-SST group. For E-SST, a 2-, 5-, and 10-year DFS rate of 58.8%, 47.1%, and 37.6% was found, compared with 62.3%, 52.9%, and 43.3% for the patients in the L-SST group (*p* = 0.79).

## Discussion

In this large uniformly staged series of patients with SST, all of whom were operated on by the same group of surgeons, the trimodality approach of induction CRT followed by surgery in medically and technically operable SST patients resulted in high locoregional control rates with acceptable 90-day mortality, even when extended surgery was needed. Survival was better in patients with an R0 resection and in those with a MPR (which was predominantly pCR) to induction therapy; however, distant metastases remain a major problem. This supports new strategies that aim to increase R0 and MPR rates and improve systemic control and highlights the importance of patients and caregivers being aware of alarming symptoms that might prompt the earlier detection and treatment of recurrent/metastatic disease.

Trimodality therapy has been a standard approach for fit patients with SST because the SWOG 9416 and JCOG 9806 trials reported good survival outcomes (5-y unselected OS 44.0% and 56.0%, respectively).^2^^,^^3^ Our real-world institutional outcomes matched/exceeded these benchmarks, despite the inclusion of patients requiring extended resection and patients with borderline operability before starting induction CRT. This might, at least in part, be explained by the following: (1) in our study, all patients were thoroughly staged and selected, undergoing PET(/CT) scan and brain imaging before CRT, and a further PET(/CT) scan after finishing induction treatment to exclude those with progression that would preclude surgery; (2) radiotherapy has evolved over the years, helping to reduce doses to surrounding organs, and compared with the SWOG/JCOG trials in which the median RT dose was 45 Gy, our patients received a median of 50Gy, perhaps contributing to the higher pCR (42% versus 36.4% and 16.0% in SWOG and JCOG, respectively); and (3) all patients were operated on by a small group of surgeons experienced in (extended) SST resection, and where necessary the team included dedicated neuro/orthopedic surgeons. By comparison, in the SWOG study, 76 surgeons operated on 110 patients entered onto the trial, an average of 1.4 patients/surgeon.

This large institutional study may function as a contemporary historical benchmark, including for the current therapeutic era, which has been largely defined by the adoption of adjuvant immunotherapy for the curative-intent nonsurgical treatment of locally advanced NSCLC. The recently published long-term survival data from the PACIFIC-trial revealed a significantly improved 5-year OS and DFS in patients with unresectable stage III NSCLC receiving adjuvant immunotherapy. These results and the promising outcomes from other trials investigating adjuvant immunotherapy for the treatment of early stage NSCLC have led to new treatment recommendations.^11^, ^12^, ^13^ Immunotherapy strategies in the setting of resectable and borderline resectable locally advanced NSCLC are currently being tested in multiple ongoing trials, several of which include patients with SST.^14^^,^^15^ Although nodal status was predominantly N0–1, more than 40% of patients in our study developed metastatic disease, even after accurate (re-)staging with FDG-PET(/CT) and brain imaging, supporting the testing of strategies aimed at improving distant control, for example, adjuvant immunotherapy after trimodality therapy.

Despite improvements in nonsurgical treatment, surgery remains relevant in SST: for patients presenting with clinical stages IIB to III in the Netherlands, the resection rate is 25%.^5^ And although 43.5% of patients with SST present with clinical stage IV, who in general are not considered for surgery, local control by means of a radical resection may be important to prevent debilitating pain if the tumor progresses or recurs locally by invading structures such as the brachial plexus and spinal cord.^5^ In addition, although the results from the PACIFIC study are promising, the need for surgery may even increase as systemic control improves, increasing the time available for local recurrence to occur. In line with the results of salvage surgery for locoregional recurrence after high-dose CRT for locally advanced NSCLC, these patients may benefit from surgical salvage once local recurrence is present.^16^ This means that in the current era of widespread enthusiasm for immunotherapy, the role of surgery in SST should not be forgotten or marginalized in training programs or when it comes to ensuring the availability of a group of highly experienced thoracic oncology surgeons.

This series includes a substantial proportion of tumors requiring extended resections (13.7%) for SST, and favorable outcomes in this population strongly suggest that surgical expertise is important for the management of SST. In multivariate Cox regression analysis, only MPR (predominantly pCR) remained significant; 88.9% of R1 resections were present in patients without an MPR which most likely explains the insignificance of R1 in multivariate analysis. At tertiary referral centers, the close cooperation between multiple surgical specialists and the availability of necessary peri-and postoperative care can ensure the best possible trimodality results with the least morbidity and mortality. We encourage the discussion of all nonmetastasized, medically operable SST patients with a center experienced in SST surgery.

We acknowledge that this study has several limitations, including the following: (1) Owing to the retrospective nature of this study, morbidity may be underreported. Accurate information on morbidity is important and should be fully discussed with patients and outweighed by the unproven possibility of additional survival when compared with the standard treatment, which is currently CRT with consolidation durvalumab. In addition, local failure in this type of tumor may result in debilitating, hard-to-treat pain, which can support aggressive surgical treatment.^17^ This is then preferably performed in the primary as opposed to salvage setting because surgery for local failure after CRT plus consolidation immunotherapy is not without risks.^18^ (2) There were a large number of patients designated as having NSCLC-NOS (41%), of which most (66.7%) were diagnosed in the early study years (2002–2010). Although an accurate histologic diagnosis may have prognostic and treatment implications,^1^ treatment decision-making was not based on pathology. (3) Although this is one of the largest series reporting long-term outcome data of patients with SST, the number of patients with extended resections (E-SST) is small, limiting robust recommendations for this group of patients.

In conclusion, for patients with SST, trimodality treatment consisting of concurrent CRT followed by surgical resection leads to good OS and DFS rates, even for patients needing extended resections. Ideally, all patients with SST should be discussed in a multidisciplinary tumor board attended by thoracic surgeons experienced in (complex) oncological resections. Distant control remains a problem and is an important focus for future research and trials, including those exploring the role of immunotherapy in the induction and adjuvant settings.

## CRediT Authorship Contribution Statement

**I. Bahce:** Writing—review and editing.

**J. A. Braun:** Writing—review and editing.

**M. Dahele:** Conceptualization; Formal analysis; Methodology; Resources; Software; Supervision; Visualization; Roles/Writing—original draft; Writing—review and editing.

**C. Dickhoff:** Conceptualization; Data curation; Formal analysis; Investigation; Methodology; Project administration; Resources; Supervision; Visualization; Roles/Writing—original draft; Writing—review and editing.

**S. Hashemi:** Writing—review and editing.

**D. J. Heineman:** Writing—review and editing.

**M. A. Paul:** Conceptualization; Data curation; Formal analysis; Methodology; Resources; Supervision; Writing—review and editing.

**S. Senan:** Conceptualization; Writing—review and editing.

**S. Ünal:** Conceptualization; Data curation; Formal analysis; Investigation; Methodology; Resources; Software; Visualization; Roles/Writing—original draft.

**M. van Dorp:** Formal analysis; Methodology; Software; Visualization; Roles/Writing—original draft.

**J. A. Winkelman:** Data curation; Resources; Roles/Writing—original draft.
