# Supplementary material for: Long-Term Outcomes After Chemoradiotherapy and Surgery for Superior Sulcus Tumors
Source: JTO Clin Res Rep. 2023 Feb 24;4(4):100475. doi: 10.1016/j.jtocrr.2023.100475 (PMC10031478; doi:10.1016/j.jtocrr.2023.100475)
Supplement: Supplementary Tables [file mmc1.docx]

**Supplementary files**

**Supplementary Table 1:** Potential predictors for survival of patients with SST treated with chemoradiotherapy and surgery

| Analysis of potential predictors for OS | | | | | | | | | |
| --- | --- | --- | --- | --- | --- | --- | --- | --- | --- |
|  |  |  | Univariate | | |  | Multivariate | | |
| Variables | *N* |  | *P* | HR | 95% CI |  | *p* | HR | 95% CI |
| Age ≥ 57 (median) | 64 |  | 0.16 | 1.43 | 0.87 – 2.36 |  |  |  |  |
| Male sex | 77 |  | 0.12 | 1.55 | 0.89 – 2.67 |  |  |  |  |
| R1 resection | 9 |  | **0.003** | 3.14 | 1.48 – 6.65 |  | 0.25 | 1.62 | 0.71 – 3.68 |
| cN positive | 40 |  | 0.13 | 1.58 | 0.95 – 2.65 |  |  |  |  |
| MPR (including pCR) | 84 |  | **<0.001** | 0.33 | 0.20 – 0.55 |  | **<0.001** | 0.37 | 0.22 – 0.65 |
| cT4 | 47 |  | 0.78 | 0.93 | 0.56 – 1.56 |  |  |  |  |
| Dose RTx (Gy) |  |  |  |  |  |  |  |  |  |
| 30-39 | 27 |  |  | 1.0 |  |  |  |  |  |
| 50-59 | 66 |  | 0.80 | 0.92 | 0.50 – 1.71 |  |  |  |  |
| 60-66 | 30 |  | 0.87 | 0.94 | 0.46 – 1.94 |  |  |  |  |
| Histopathology |  |  |  |  |  |  |  |  |  |
| NSCLC-NOS | 51 |  |  | 1.0 |  |  |  |  |  |
| Adenocarcinoma | 42 |  | 0.80 | 1.08 | 0.60 - 1.94 |  |  |  |  |
| Squamous cell carcinoma | 30 |  | 0.65 | 0.86 | 0.45 – 1.66 |  |  |  |  |
| Analysis of potential predictors for DFS | | | | | | | | | |
| Age ≥ 57 (median) | 64 |  | 0.23 |  |  |  |  |  |  |
| Male sex | 77 |  | 0.27 |  |  |  |  |  |  |
| R1 resection | 9 |  | **0.003** | 3.06 | 1.45 – 6.44 |  | 0.25 | 1.60 | 0.72 – 3.56 |
| cN positive | 40 |  | 0.32 |  |  |  |  |  |  |
| MPR | 84 |  | **<0.001** | 0.33 | 0.21 - 0.54 |  | **<0.001** | 0.37 | 0.22 - 0.61 |
| cT4 | 47 |  | 0.40 | 0.81 | 0.49 – 1.33 |  |  |  |  |
| Dose RTx (Gy) |  |  |  |  |  |  |  |  |  |
| 30-39 | 27 |  |  | 1.0 |  |  |  |  |  |
| 50-59 | 66 |  | 0.80 | 0.93 | 0.52 – 1.66 |  |  |  |  |
| 60-66 | 30 |  | 0.90 | 1.05 | 0.53 – 2.00 |  |  |  |  |
| Type of pathology |  |  |  |  |  |  |  |  |  |
| NSCLC-NOS | 51 |  |  | 1.0 |  |  |  |  |  |
| Adenocarcinoma | 42 |  | 0.78 | 1.09 | 0.63 – 1.86 |  |  |  |  |
| Squamous cell carcinoma | 30 |  | 0.57 | 0.83 | 0.44 – 1.57 |  |  |  |  |

R1 = Microscopically incomplete resection, cN = node positive at clinical staging, MPR = major pathologic response, pCR = pathologic complete response, RTx = Radiotherapy, NSCLC-NOS = Non-small cell lung cancer not otherwise specified

**Supplementary Table 2:** Comparison of characteristics between E-SST ( n=17) and L-SST (n=106)

|  | **E-SST** | **L-SST** | **p-value** |
| --- | --- | --- | --- |
| **Characteristics** | | |  |
| Age (mean, SD) | 51 (9.0) | 57 (9.3) | 0.01 |
| Involvement of lymph nodes at baseline staging | 2 (11.8%) | 38 (35.8%) | 0.049 |
| R0 resection | 16 (94.1%) | 98 (92.5%) | 1.0 |
| Major pathologic response | 13 (76.5%) | 71 (57.7%) | 0.44 |
| Clinical T status  T4 | 14 (82.4%) | 33 (30.8%) | <0.001 |
